# Nitrogen enrichment alters nutrient resorption and exacerbates phosphorus limitation in the desert shrub *Artemisia ordosica*

**DOI:** 10.1002/ece3.4407

**Published:** 2018-09-24

**Authors:** Jing Zheng, Weiwei She, Yuqing Zhang, Yuxuan Bai, Shugao Qin, Bin Wu

**Affiliations:** ^1^ School of Soil and Water Conservation Beijing Forestry University Beijing China; ^2^ Key Laboratory of State Forestry Administration on Soil and Water Conservation Beijing Forestry University Beijing China; ^3^ Engineering Research Center of Forestry Ecological Engineering, Ministry of Education Beijing Forestry University Beijing China

**Keywords:** *Artemisia ordosica*, desert ecosystem, global environmental changes, nitrogen deposition, nutrient limitation, nutrient resorption

## Abstract

Increasing nitrogen (N) deposition and precipitation are major drivers of global changes that are expected to influence plant nutrient resorption in desert ecosystems, where plant growth is often nutrient and water limited. However, knowledge on the effects of increased N and precipitation on them remain poorly understood. This study determined the effects of increased N (ambient, 60 kg N ha^−1^ year^−1^) and water supply (ambient, +20%, +40%), and their combination on the leaf nutrient resorption of *Artemisia ordosica*, a dominant shrub in the Mu Us Desert of northern China. After 2 years of treatments, only N addition significantly decreased the N resorption efficiency of *A. ordosica*. Both N and water addition had no effect on the phosphorus (P) resorption efficiency of this shrub, and there were no interactive effects of N and water availability on shrub nutrient resorption. The responses of shrub leaf N:P ratio tended to saturate as soil available N:P increased. The aboveground net primary productivity of *A. ordosica* was positively correlated with leaf P resorption efficiency, rather than N resorption efficiency. Our results suggest that N addition exacerbated the P limitation of the shrub growth and played a more fundamental role than water addition in controlling the nutrient resorption process of the desert shrub *A. ordosica*. This information contributes to understand the relationship between nutrient conservation strategy and plant growth of desert shrub species under global environmental changes.

## 1 INTRODUCTION

Global environmental changes, including widespread nitrogen enrichment and altered precipitation patterns, are predicted to occur in the future (Galloway et al., 2008; IPCC, 2013). An increase in both atmospheric N deposition (Liu et al., 2013) and precipitation (Intergovernmental Panel on Climate Change (IPCC), 2013) is projected to occur in the desert areas of China in the coming decades. These changes are expected to fundamentally influence plant growth in this area, where water and nutrients are usually limited (Bobbink et al., 2010; Maestre et al., 2016). Plants in these ecosystems usually depend strongly on internal nutrient cycling and are thought to have relatively high leaf nutrient concentration and nutrient resorption to better adapt to arid environments (Killingbeck, 1993, 1996). Nutrient resorption from senescing tissues is a critical physiological process for nutrient conservation that makes plants less dependent on external nutrient availability and is, therefore, important for plant growth, survival, and adaptive ability in oligotrophic ecosystems (Aerts & Chapin, 2000; Killingbeck, 1993; Wang, Wang, He, An, & Xu, 2017). Therefore, knowledge on how nutrient resorption respond to concurrent alterations in N and water supply is important for understanding the nutrient conservation strategy of desert plants under future global environmental changes.

Nitrogen deposition and precipitation changes have considerable impacts on plant nutrient resorption processes (Huang, Su, Mu, & Li, 2018; Kou et al., 2017; Lü & Han, 2010; Ren et al., 2015; van Heerwaarden, Toet, & Aerts, 2003b; Yuan & Chen, 2015). Increased soil nutrient availability caused by N addition generally reduces plant N resorption (Li, Zheng, Han, Zheng, & Li, 2010; Li et al., 2015; Li, Gao, et al., 2016 Lü & Han, 2010; Lü, Reed, Yu, & Han, 2016; Lü et al., 2013; Zhang, Li, et al., 2015). In addition, it was gradually recognized that increased N input could cause a shift from N‐limited toward P‐limited or N and P colimitation ecosystems (Huang et al., 2016), which may lead to a more conservative use of P (i.e., higher P resorption) (Li et al., 2015). However, empirical studies of changes in plant P resorption in response to N addition still appear inconsistent, with positive (Li, Gao, et al., 2016; Lü & Han, 2010; Shen et al., 2018) and negative (Lü et al., 2013, 2016) changes and neutral effects (Kou et al., 2017; Lü, Cui, Wang, & Han, 2011) having been recorded. Such complex responses may be related to the soil available P condition or whether the ecosystem is subject to P limitation, or both (Güsewell, 2005; Lü & Han, 2010; Lü et al., 2013). To our knowledge, desert ecosystems are also P‐limited (Han, Tang, Chen, & Fang, 2013); however, little is known about the P resorption of desert plants in response to N addition.

Similar to that of N addition, it is generally hypothesized that water addition can stimulate soil microbial activity and, thus, enhance soil N availability (Liu, Zhang, & Wan, 2009; Wang, Wan, Xing, Zhang, & Han, 2006; Wang et al., 2016), which could reduce plant N resorption but increase P resorption. To date, this hypothesis has been verified in many grassland (Lü & Han, 2010; Oyarzabal, Paruelo, del Pino, Oesterheld, & Lauenroth, 2007; Zhao et al., 2017) and forest ecosystems (Oleksyn, Reich, Zytkowiak, Karolewski, & Tjoelker, 2003). A recent study conducted in a desert ecosystem found contrasting results, whereby water addition had no impacts on leaf nutrient resorption (Huang et al., 2018), suggesting that the response of plant nutrient resorption to water addition in desert ecosystems may be distinct from that in other ecosystems. Moreover, grassland studies have shown that varying plant nutrient resorption in response to changes in N availability could be mediated by soil water availability (Lü & Han, 2010; Ren et al., 2015). However, few studies have investigated these nutrient resorption responses to simultaneous and interacting N availability and water changes in desert ecosystems (but see Huang et al., 2018).

With respect to a plant's production and growth, nutrient resorption can be as important of a nutrient source as the uptake from soils (Brant & Chen, 2015; May & Killingbeck, 1992). It has been postulated that high nutrient resorption efficiency can increase plant fitness, especially in nutrient‐poor environments (Aerts, 1996; Eckstein, Karlsson, & Weih, 1999). However, there exists some controversy on the relationships between plant growth and nutrient resorption in field experiments (Lamaze, Pasche, & Pornon, 2003; May & Killingbeck, 1992; Ren et al., 2015). For instance, Zhang, Zhang, Chen, Zhang, and Poorter (2015) found that plant N resorption efficiency was positively corrected with plant growth in a forest ecosystem, suggesting that N is a key limiting factor for plant growth. In contrast, some studies showed that there were no relationships between plant growth and nutrient resorption (e.g., Eckstein, Karlsson, & Weih, 1998; Pasche, Pornon, & Lamaze, 2002). These inconsistences are probably related to the nutrient limitation of plant growth (Güsewell, 2005; Yan et al., 2015). It is easy to understand that, if a certain nutrient (e.g., N or P) was the limiting factor for growth, plants tend to increase their nutrient resorption with growth, resulting in a positive relationship between plant growth and nutrient resorption. Thus, the relationship between plant growth and nutrient resorption may depend on whether plant is limited by a nutrient, especially N and P.

To examine the responses of desert plant nutrient resorption patterns to N and water addition, we examined green and senesced leaf N and P concentrations and plant growth (i.e., aboveground net primary productivity (ANPP)) of the dominant shrub *Artemisia ordosica* Krasch with increased N and water addition in a desert shrubland in the Mu Us Desert of northern China. We hypothesized that (a) N and water addition would decrease N resorption; but (b) increase P resorption, as plants may suffer P shortage under the amendment of N and water; and (c) given that increased nutrient resorption can increase plant fitness in low‐nutrient environments, plant growth may be positively associated with P resorption efficiency, rather than N resorption efficiency.

## 2 MATERIALS AND METHODS

### 2.1 Study site

The study was conducted at the Yanchi Research Station (37°04′ to 38°10′ N and 106°30′ to 107°41′ E, 1,530 m above sea level) in Ningxia Province, northwestern China. This area has a mid‐temperate and semi‐arid continental climate and lies on the southwest edge of the Mu Us Desert. The mean annual temperature is 8.1°C and the mean annual precipitation is 284.8 mm (1955–2013), with 83.3% of rainfall occurring in the growing season from May to September. The soil type is classified as quartisamment (US Soil Taxonomy; Gao et al., 2014). The shrubland vegetation is dominated by *A. ordosica*, with sparse shrub species *Salix psammophila*,* Hedysarum mongolicum*,* Caragana korshinskii*, and grass *Agropyron cristatum*. *Artemisia ordosica*, the investigated species in our study, is a deciduous, multistemmed, dwarf shrub with plumose, linearly lobate leaves and branch system consisting of old brown branches and purple current‐year twigs (She et al., 2017). It is characterized by a deep taproot system that can reach a depth of 1–3 m, while the lateral roots are mainly distributed in the upper soil layer (0–30 cm) (Li, Yu, Werger, Dong, & Zuidema, 2011). The horizontal distribution of *A. ordosica* roots, based on root mass, is limited to a range of 0.4 m from the trunk (Zhang et al., 2008). It starts to expand leaves in early April and completes leaf abscission in mid‐November.

### 2.2 Experimental design

Our experiment was design followed that described in She, Zhang, Qin, Wu, and Bai (2016), with minor adjustments as follows. In October 2014, four experimental blocks, each containing six 5 m × 5 m plots, were established in an *A. ordosica* shrubland. Two nitrogen treatments (N0: no additional nitrogen; N60: 60 kg N ha^−1^ year^−1^) and three water addition treatments (W0: ambient; W20: ambient +20%; and W40: ambient +40%) were applied to the plots in a fully factorial design, with four replicates of each treatment combination resulting in a total of 24 plots. Each plot was arranged in a randomized block design and separated by a 1 m buffer. In the control plots, the coverage of shrubs and herbaceous plants were approximately 45%–50% and 10%–20%, respectively. The N and water treatments were carried out over 2015 and 2016.

The magnitude of water addition treatments over the two experimental years was based on the long‐term mean annual precipitation records from 1955 to 2013 (284.3 mm). Water was applied with a sprinkler irrigation system nine times in equal amounts throughout the growing season (May–September) with three times occurring in July and August and one time each in May, June, and September. In total, the amount of water added was approximately 56 and 112 mm per year in the W20 and W40 treatments, respectively. Water was always applied after a natural rainfall event to prevent altering the patterns of current‐year precipitation. In this study, we defined annual precipitation as the water‐year precipitation received between October 1 and the following September 30. The ambient water‐year precipitation was 368.9 mm in October 2015–September 2016.

For nitrogen treatments, N was supplied in solution form as NH_4_NO_3_ and applied to plots as five equal applications at the beginning of each month during the growing season. During each application, 85.71 g NH_4_NO_3_ (an analytical reagent) was weighted and dissolved in 10 L tap water. Control plots also received the same amount of water, equivalent to 2 mm precipitation, without added N. Background N deposition in the study site was about 12 kg N ha^−1^ year^−1^ according to our monitoring data (She et al., 2016). The mean N deposition rate in northern China was 56.2 kg N ha^−1^ year^−1^ (Xu et al., 2015). Nitrogen addition plots received nitrogen at rate of 60 kg N ha^−1^ year^−1^, an amount selected to simulate areas that have received high deposition. Detailed information on the dates of N and water addition is shown in Supporting Information Figure S1.

### 2.3 Field sampling and measurements

In July 2016, five *A. ordosica* individuals were randomly selected in each plot; however, selections were deliberately made away from plot edges. Four twigs with a length of about 30 cm were marked with plastic labels for each individual. We harvested the fully expanded sun leaves from two of the four marked twigs per individual in early September (the peak of the growing season). The remaining leaves from the other twigs were monitored weekly from late September to November and collected only when they were fully senesced. Senescing leaves are dry and brown, and can be detached by gently flicking the branch or leaf. The leaf samples from each individual were pooled as a mixed sample in each plot. All plant samples (both green and senesced leaves) were oven‐dried at 75°C for 48 hr, then ground and stored for chemical analysis. Shrub (*A. ordosica*) ANPP (g/m^2^) was estimated by a nondestructive method based on twig size and number, following the methodologies outlined in She et al. (2016).

Total N concentration was analyzed using an elemental analyzer (Vario EL III, CHNOS Elemental Analyzer, Elementar Analysensysteme GmbH, Germany). Total P concentration was measured using the Mo‐Sb colorimetric method. Both N and P concentrations were expressed on a mass basis.

Soil samples (0–20 cm depth) were collected for each plot in mid‐September 2016. In each plot, three soil cores were randomly collected from open areas between shrubs with using a 3.8 cm diameter soil auger and were mixed into a single composite sample. All soil samples were sieved through a 2 mm mesh to remove the roots. Fresh soil samples were extracted using 2 M KCl solution and the ammonium and nitrate concentrations in the filtered extract were determined by spectrophotometric method. Gravimetric water content was determined by drying at 105°C for 24 hr. Available soil P was estimated by extracting air‐dried soil with 0.5 M NaHCO_3_ and using the molybdenum blue‐ascorbic acid method (Olsen, Cole, Watanabe, & Dean, 1954).

### 2.4 Calculation and statistical analysis

The nutrient resorption efficiency (N_u_RE) (Zhao et al., 2017), defined as the proportion of the mature leaf nutrient pool that is resorbed, was calculated as:

$$N_{u}\text{RE}\left(\%\right)\;=\;\left[\frac{N_{\mathrm{gr}}-N_{\mathrm{sen}}}{N_{\mathrm{gr}}}\right]\;\;\times \;100\%$$

where N_gr_ and N_sen_ are the concentrations of N or P from green and senesced leaves of *A. ordosica* in each plot, respectively.

All the data met the assumption of normality using the Shapiro–Wilk normality test. Therefore, we used untransformed data for statistical analysis. Two‐way ANOVAs was used to detect the main and interactive effects of N and water addition on soil moisture, inorganic N, and available P, leaf nutrient concentrations, leaf N:P ratio, and nutrient resorption efficiency. One‐way ANOVAs with Duncan's multiple range tests were performed to compare the N effects on each response variable at each water‐treatment level and the watering effects at each N addition rate. Then, to examine the relationship pattern between leaf N:P and soil available N:P, we compared two types of model, linear (*y* = a + b* *x*) and logarithmic [*y* = a + b* log(*x*)], with Akaike information criterion (AIC) as criterion. Finally, we used linear models to test the relationships between leaf nutrient resorption efficiency (%) and shrub ANPP (g/m^2^) or soil available nutrient concentrations (mg/kg). For all analyses, statistical significance was determined at a level of *p *≤* *0.05. All analyses and figures were carried out using R version 3.3.1 (R Core Team 2016). We used the package ggplot2 (Wickham, 2016) to produce graphical data representations.

## 3 RESULTS

**Figure 1:**
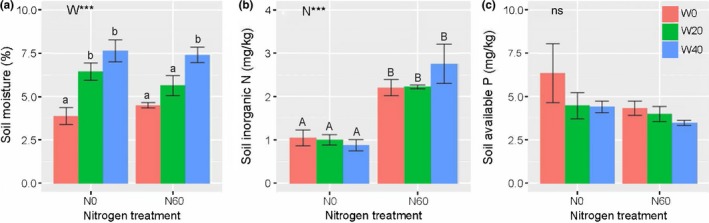
Effects of N and water addition on (a) soil moisture, (b) inorganic N, and (c) available P. Data are means ± *SE*, where sample size is four in all treatments. ***p <* *0.001; while *ns* indicates nonsignificance. The different capital letters indicate that significant differences (*p *<* *0.05) among N treatments. The different lowercase letters indicate that significant differences (*p *<* *0.05) among water treatments

Nitrogen and water addition had nonsignificant effects on soil available P, but had remarkable effects on soil moisture and inorganic N concentration (Figure 1, Table 1). Soil moisture significantly increased only in plots with added water, from 4.18 ± 0.27 (W0) to 6.04 ± 0.38 (W20) and 7.53 ± 0.36% (W40), an increase rate of 44.50% and 80.14% on average, respectively (*p *<* *0.05, Figure 1a). Soil inorganic N concentrations significantly increased only in plots with added N, from 0.98 ± 0.08 (N0) to 2.40 ± 0.17 mg/kg (N60), a dramatic increase rate of 144.90% on average (*p *<* *0.05, Figure 1b).

**Table 1:** Results of two‐way ANOVAs for soil moisture (SM), inorganic nitrogen (In‐N), and available phosphorus (Av‐P) concentration as dependent on nitrogen addition (N), water addition (W), and their interaction (N × W)

	SM	In‐N	Av‐P
N	0.14	49.33***	2.56
W	25.77***	0.41	1.41
N × W	1.19	1.27	0.42

Note

The F‐ratios are presented, together with their level of significance.

***p <* *0.001.

**Figure 2:**
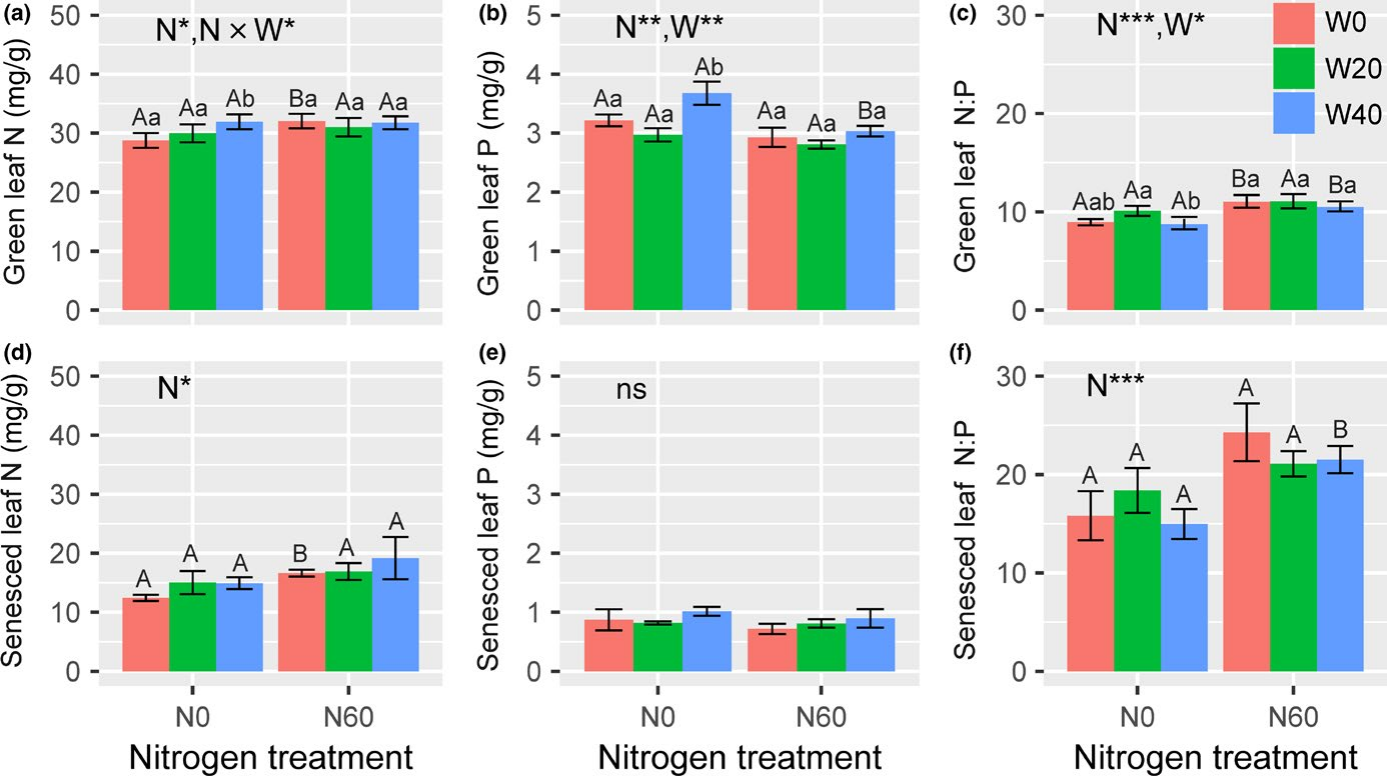
Effects of N and water addition on (a, c) N and (b, d) P concentrations in green and senesced leaves of *A. ordosica*. Data are means ± *SE*, where sample size is four in all treatments. *p <* *0.05; **p <* *0.01; while *ns* indicates nonsignificance. The different capital letters indicate that significant differences (*p *<* *0.05) among N treatments. The different lowercase letters indicate that significant differences (*p *<* *0.05) among water treatments

Nitrogen addition significantly enhanced both green and senesced leaf N concentrations (Table 2), from 30.21 ± 0.80 and 14.12 ± 0.77 mg/g (N0) to 31.60 ± 0.70 and 17.55 ± 1.23 mg/g (N60), an increase rate of 4.60% and 24.29% on average, respectively (*p *<* *0.05, Figure 2a,d). There was a significant interaction between N and water addition affecting green leaf N concentrations (*p *<* *0.05, Table 2, Figure 2a), where increases in the green leaf N concentrations induced by water addition were more significant in the unfertilized (Duncan's test, *p *<* *0.05) than that in the fertilized plots (Duncan's test, *p *>* *0.05). Surprisingly, N‐induced increases in the green leaf N concentrations were found in the ambient plots (W0, Duncan's test, *p *<* *0.05), but not in the watered plots (W20 and W40, Duncan's test, *p *>* *0.05). Nitrogen and water addition had opposite effects on green leaf P concentrations (Table 2; Figure 2b). Nitrogen addition (*p *<* *0.05) decreased but water addition (*p *<* *0.05) increased P concentrations in green leaves, and no interactions were found (Figure 2b). Nitrogen‐induced decreases in green leaf P concentrations were 11.24% in the fertilized plots (N60, 2.92 ± 0.07 mg/g) lower than in unfertilized plots (N0, 3.29 ± 0.12 mg/g). Watering increased green leaf P concentrations from 3.07 ± 0.10 mg/g (W0) and 2.89 ± 0.07 mg/g (W20) to 3.36 ± 0.16 mg/g (W40), an increase rate of 9.45% and 16.26% on average, respectively. Neither N addition nor water addition, nor their interaction, affected P concentrations in senesced leaves (Figure 2e). Furthermore, N addition significantly increased green and senesced leaf N:P ratios (Table 2), from 9.27 ± 0.30 and 16.39 ± 1.20 (N0) to 10.86 ± 0.32 and 22.31 ± 1.13 (N60), an increase rate of 17.15% and 36.12% on average, respectively (*p *<* *0.05, Figure 2c,f). However, water addition only significantly decreased green leaf N:P ratio (Figure 2f), from 9.98 ± 0.49 (W0) to 9.62 ± 0.46 (W40), a little decrease rate of 3.61% on average.

**Table 2:** Results of two‐way ANOVAs for leaf nutrient variables and nutrient resorption parameters as dependent on nitrogen addition (N), water addition (W), and their interaction (N × W)

	[N]g	[N]s	[P]g	[P]s	NRE	PRE	[N:P]g	[N:P]s
N	7.54*	8.40*	11.98**	1.61	5.82*	0.003	30.41***	38.78***
W	3.35	1.50	6.62**	1.83	0.91	0.32	3.89*	1.37
N × W	3.97*	0.43	1.90	0.34	0.40	0.20	1.27	3.17

Note

The F‐ratios are presented, together with their level of significance. Ng and Pg represent N and P content in green leaves, respectively; Ns and Ps represent N and P content in senesced leaves, respectively; N:Pg and N:Ps represent the ratios of green and senesced leaf nitrogen and phosphorus, respectively; and NRE and PRE represent N and P resorption efficiency, respectively.

*p <* *0.05; **p <* *0.01; ***p <* *0.001.

**Figure 3:**
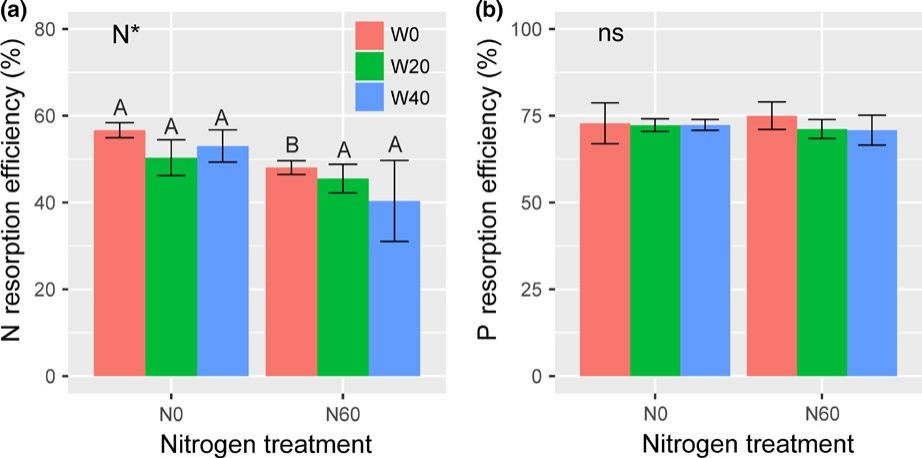
Effects of N and water addition on (a) N and (b) P resorption efficiency of *A. ordosica*. Data are means ± *SE*, where sample size is four in all treatments. *p <* *0.05; while *ns* indicates nonsignificance. The different capital letters indicate that significant differences (*p *<* *0.05) among N treatments

**Figure 4:**
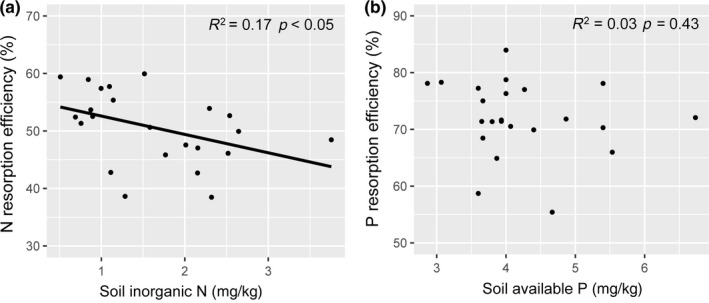
Relationships between (a) soil inorganic N concentration and N resorption efficiency and (b) between soil available P and P resorption efficiency. Values greater than triple standard deviations from the mean were deemed abnormal, resulting in one abnormal observation being removed prior to regression analysis in the left column (a) and right column (b), respectively

In the ambient plots, the average N resorption efficiency (NRE) and P resorption efficiency (PRE) were 56.67 ± 1.73% and 72.81 ± 5.90%, respectively. Nitrogen treatment significantly reduced NRE, but watering and their interaction had no effects (Table 2). Nitrogen‐induced decrease in NRE in the fertilized plots (N60, 44.65 ± 3.17%) were 16.31% lower than that in the unfertilized plots (N0, 53.35 ± 1.92%) on average (Figure 3a). Nitrogen, water addition, and their interaction had nonsignificant effects on PRE (Table 2). In addition, NRE was negatively correlated with soil inorganic N concentrations (*p *<* *0.05, Figure 4a). There was no significant relationship between PRE and soil available P concentrations (*p *>* *0.05, Figure 4b).

**Figure 5:**
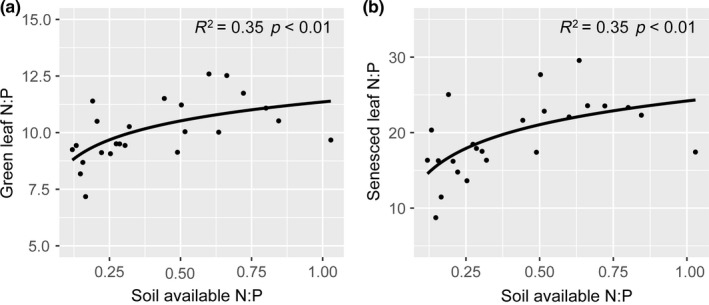
Relationships between soil available N:P and green (a) and senesced (b) leaf N:P

**Figure 6:**
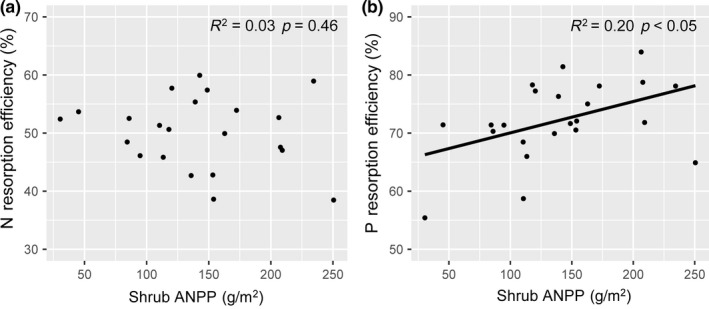
Relationships between the shrub ANPP and leaf NRE (a) and PRE (b). Values greater than triple standard deviations from the mean were deemed abnormal, resulting in two abnormal observations being removed prior to regression analysis in the left column (a) and one abnormal observation being removed prior to regression analysis in the right column (b)

Both the linear model and the logarithmic model could be significantly fitted to the relationship between soil available N:P and green/senesced leaf N:P (green leaf: Linear: *R* ^2^ = 0.27, *p *<* *0.01, Logarithmic: *R* ^2^ = 0.35, *p *<* *0.01; senesced leaf: Linear: *R* ^2^ = 0.28, *p *<* *0.01, Logarithmic: *R* ^2^ = 0.35, *p *<* *0.01). The logarithmic model was a better fit than the linear model as indicated by AIC (green leaf: AIC_linear_ = 79.15, AIC_logarithmic_ = 76.47; senesced leaf: AIC_linear_ = 142.17, AIC_logarithmic_ = 139.80) (Supporting Information Table S1; Figure 5). The ANPP of shrubs was not associated with leaf NRE (*R* ^2^ = 0.03, *p *=* *0.46, Figure 6a), but was significantly and positively related to leaf PRE (*R* ^2^ = 0.20, *p *<* *0.05, Figure 6b).

## 4 DISCUSSION

Our data partly supported the first hypothesis and showed that N addition significantly decreased leaf NRE, while water addition had no effect on NRE. Such a decrease in the NRE of *A. ordosica* probably resulted from the increased soil N availability following N addition (Figure 4a). Similarly, N addition has been reported to reduce plant N resorption in several ecosystems worldwide, including grasslands (Li et al., 2015; Li, Gao, et al., 2016; Lü & Han, 2010; Lü et al., 2013; Wang, Xu, et al., 2017), forests (Li et al., 2010), subarctic peat bogs (van Heerwaarden et al., 2003b), and temperate shrublands (Zhang, Li, et al., 2015), suggesting that an increase in N supply could enhance the plants’ uptake of N from the external environment and, therefore, reduce dependency on internal N recycling (Li, Gao, et al., 2016; Lü et al., 2013). It is worth noting that we only used mass‐based method to estimate the nutrient resorption, which may result in considerable underestimation of calculating NRE (van Heerwaarden, Toet, & Aerts, 2003a). However, it is not expected to hide the response trends to N addition (Huang et al., 2012; van Heerwaarden et al., 2003b). Surprisingly, there were no significant effects of water addition on NRE in this study. This result was not consistent with findings from other field studies in semi‐arid grasslands (Lü & Han, 2010; Ren et al., 2015), in which “fertilization effects” caused by water addition can decrease NRE. A study based on a global dataset also showed that NRE are negatively related to mean annual precipitation (Yuan & Chen, 2009), suggesting that N resorption may be regulated by precipitation. The nonsignificant water effects in our study were attributed to the finding that soil N availability was not affected by water addition (Figure 1b). Because the desert soil in our study contained only a small amount of organic matter (soil organic carbon content, 2.88 ± 0.18 g/kg; She et al., 2018), it is likely that water addition could not be enough to mineralize more available N from organic matter and thus could not enhance soil N availability (Wang et al., 2006). Alternatively, the simultaneous water addition with high natural precipitation in the present study may have led to soil N loss through leaching and denitrification (Huang, Yu, Li, Yuan, & Bartels, 2009; Srivastava, Singh, Tripathi, Singh, & Raghubanshi, 2018), resulting in no change in soil inorganic N availability. A recent study conducted in a temperate desert supports our findings, in which water addition had no impacts on leaf NRE of desert shrubs (Huang et al., 2018). Together, these results indicate that water addition effects on plant N resorption efficiency are dependent on soil resource condition (e.g., organic matter content) and climatic factors (wet or dry year). In contrast with the findings of the grassland ecosystem studies (Lü & Han, 2010; Ren et al., 2015), our results showed that there was no interactive effect of N and water availability on the NRE, indicating that the response of the internal plant N resorption processes to N addition might not be mediated by soil water availability in the desert ecosystem.

In contrast to the NRE pattern, neither N, water addition, nor their interaction had an effect on PRE of *A. ordosica*. These findings differed from our hypothesis in which N and water addition were expected to increase PRE. A previous study also showed a noneffect of increased N and water addition on PRE in a desert ecosystem (Huang et al., 2018). These results can be explained by the fact that the extremely low availability of P (6.37 mg/kg) in the soils and the very high (>72%) PRE of *A. ordosica* in the ambient plots, which could indicate that this shrub was more likely to adopt near complete utilization of P from the soil and senesced leaves under natural conditions (Killingbeck, 1996). Consequently, the shrub in this study would not be able to extract additional P from senescing leaves, despite N and water addition. Such low soil available P and high P resorption efficiency also suggest that the shrub growth could be largely limited by P. Moreover, the saturated response of leaf N:P ratio to soil available N:P ratio (Figure 5), imply that N addition could further exacerbate P limitation of the shrub growth. Collectively, our results suggest that the shrub *A. ordosica* probably have almost resorbed P from the senesced leaves under natural condition, resulting in noneffect of N and water addition on leaf PRE.

Our findings support our hypothesis that plant growth was positively associated with P resorption efficiency, rather than N resorption efficiency. Our findings suggested that the higher the growth of *A. ordosica*, the more P is needed to be resorbed from senesced leaves. It is well known that the most limiting factor determines the plant growth (van der Ploeg, Böhm, & Kirkham, 1999). Nitrogen addition could lead to an alleviation of N limitation (Lü et al., 2013), resulting in a decrease in NRE and a nonsignificant relationship between plant growth and NRE. In contrast, increased N enrichment is likely to exacerbate P limitation of plant growth (Huang et al., 2016; Li, Niu, & Yu, 2016; Zhan, Wang, Zhu, Li, & Bai, 2017), which probably contributes to the positive correlation between plant growth and PRE. Collectively, these results suggest that N addition could alleviate N limitation but exacerbate P limitation, leading to a shrub growth that is associated with PRE but not with NRE in this desert ecosystem.

## 5 CONCLUSIONS

In summary, our results showed that the N resorption efficiency of *A. ordosica* significantly decreased following N enrichment, while water addition had no substantial effects. Neither N enrichment nor water addition had significant influences on the shrub P resorption efficiency. There was a nonsignificant interaction between N and water addition affecting leaf nutrient resorption. These results imply that the nutrient resorption of *A. ordosica* was mainly affected by N addition, rather than water addition. The ANPP of *A. ordosica* was positively correlated with leaf P resorption efficiency, together with the saturated response of leaf N:P ratio to soil available N:P ratio, suggesting that N addition could exacerbate P limitation of the shrub growth in this desert ecosystem.

## CONFLICT OF INTEREST

None declared.

## AUTHORS’ CONTRIBUTIONS

Jing Zheng, Yuqing Zhang, and Bin Wu conceived the ideas and designed the study; Weiwei She, Yuxuan Bai, and Shugao Qin performed the data collection and analysis; Jing Zheng and Weiwei She interpreted the results and wrote the manuscript.

## DATA ACCESSIBILITY

Data are available on the Dryad Digital Repository (https://doi.org/10.5061/dryad.4r1435g).
