# Supplementary material for: Nitrogen enrichment alters nutrient resorption and exacerbates phosphorus limitation in the desert shrub Artemisia ordosica
Source: Ecol Evol. 2018 Sep 24;8(20):9998–10007. doi: 10.1002/ece3.4407 (PMC6206216; doi:10.1002/ece3.4407)

Supplementary Material

**Nitrogen enrichment alters nutrient resorption and exacerbates phosphorus limitation in the desert shrub *Artemisia ordosica***

**Jing Zheng ^1^, Weiwei She ^1^, Yuqing Zhang ^1, 2, *^, Yuxuan Bai ^1^, Shugao Qin ^1, 3^, Bin Wu ^1, 2^**

*** Correspondence:** Yuqing Zhang: zhangyqbjfu@gmail.com

**Table S1.** The regressions between [N:P]g and [N:P]s with soil available N:P ratio as independent variable. Linear: y = a + b* x; Logarithmic: Y = a + b* log(x)

| **Dependent Variable** | **Model** | **Parameter Estimates** | | | ***R*^2^** | | ***P*** | | ***AIC*** | ***△AIC*** | |
| --- | --- | --- | --- | --- | --- | --- | --- | --- | --- | --- | --- |
|  |  | **a** | **b** | |  |  |  |  |  |  |  |
| [N:P]g | Linear | 8.95 | | 2.67 | | 0.27 | | <0.01 | 79.15 | | 2.68 |
|  | **logarithmic** | **11.35** | | **1.21** | | **0.35** | | **<0.01** | **76.47** | | **0** |
| [N:P]s | Linear | 15.08 | | 10.23 | | 0.28 | | <0.01 | 142.17 | | 2.37 |
|  | **logarithmic** | **24.20** | | **4.54** | | **0.35** | | **<0.01** | **139.80** | | **0** |

[N:P]g and [N:P]s represent the ratios of green and senesced leaf nitrogen and phosphorus, respectively.





**Figure S1.** Temporal trends of precipitation in the 2016 growing season. The solid lines indicate precipitation events. The different color lines represent the dates of N addition, water addition, soil and leaf sampling.

**
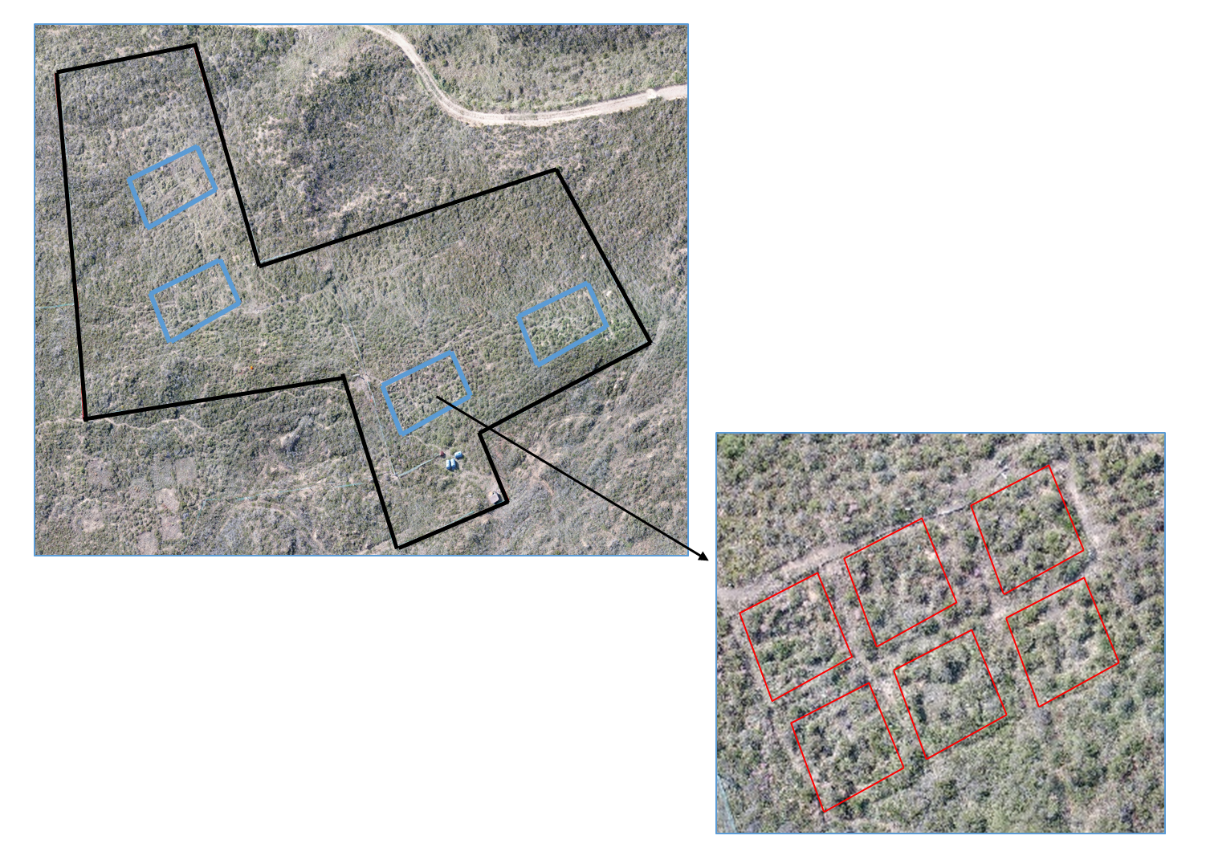
**

**Figure S2.** The distribution of experimental plots. The blue regions indicate four experimental blocks, and the red regions indicate six experimental plots in a block.


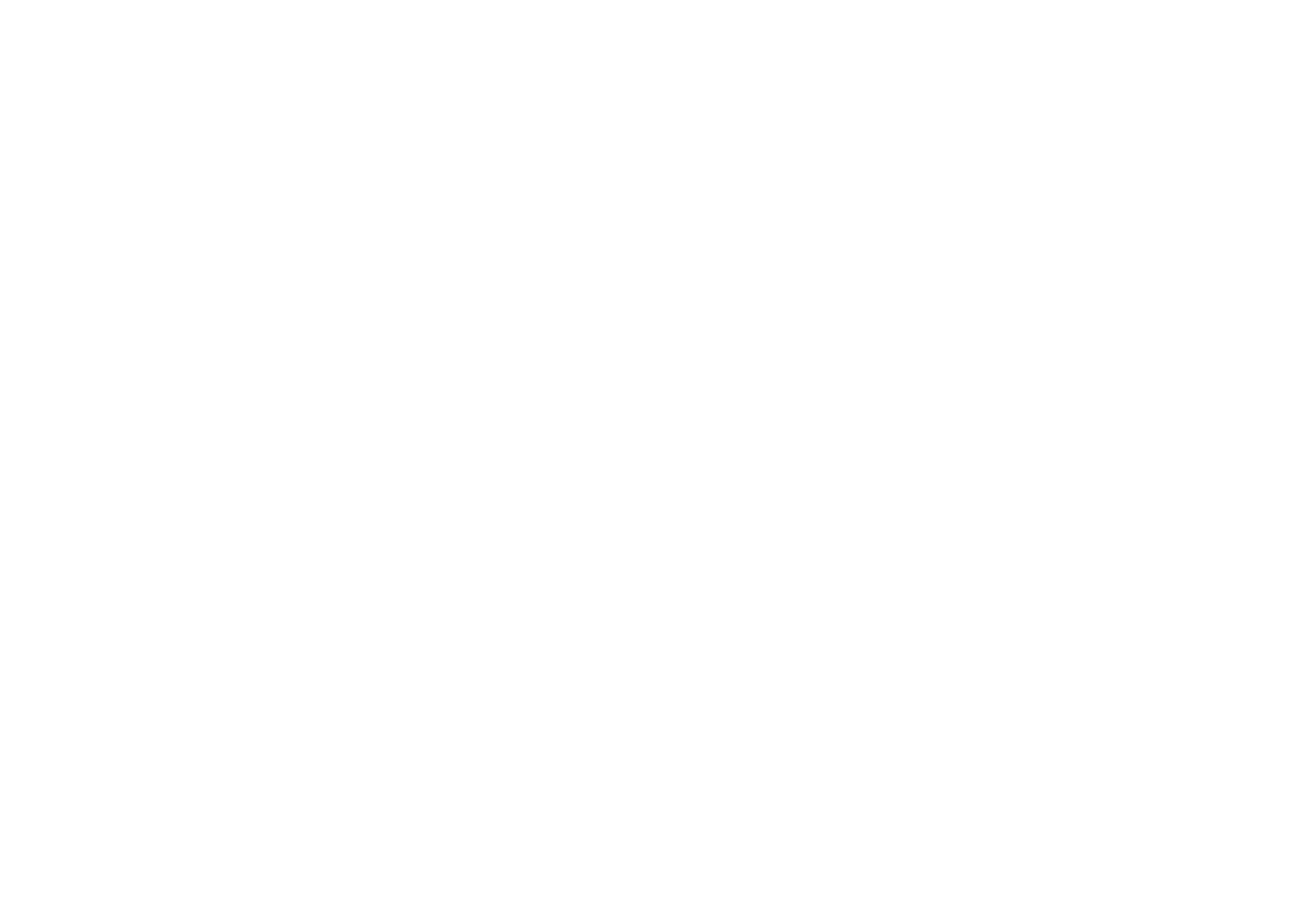


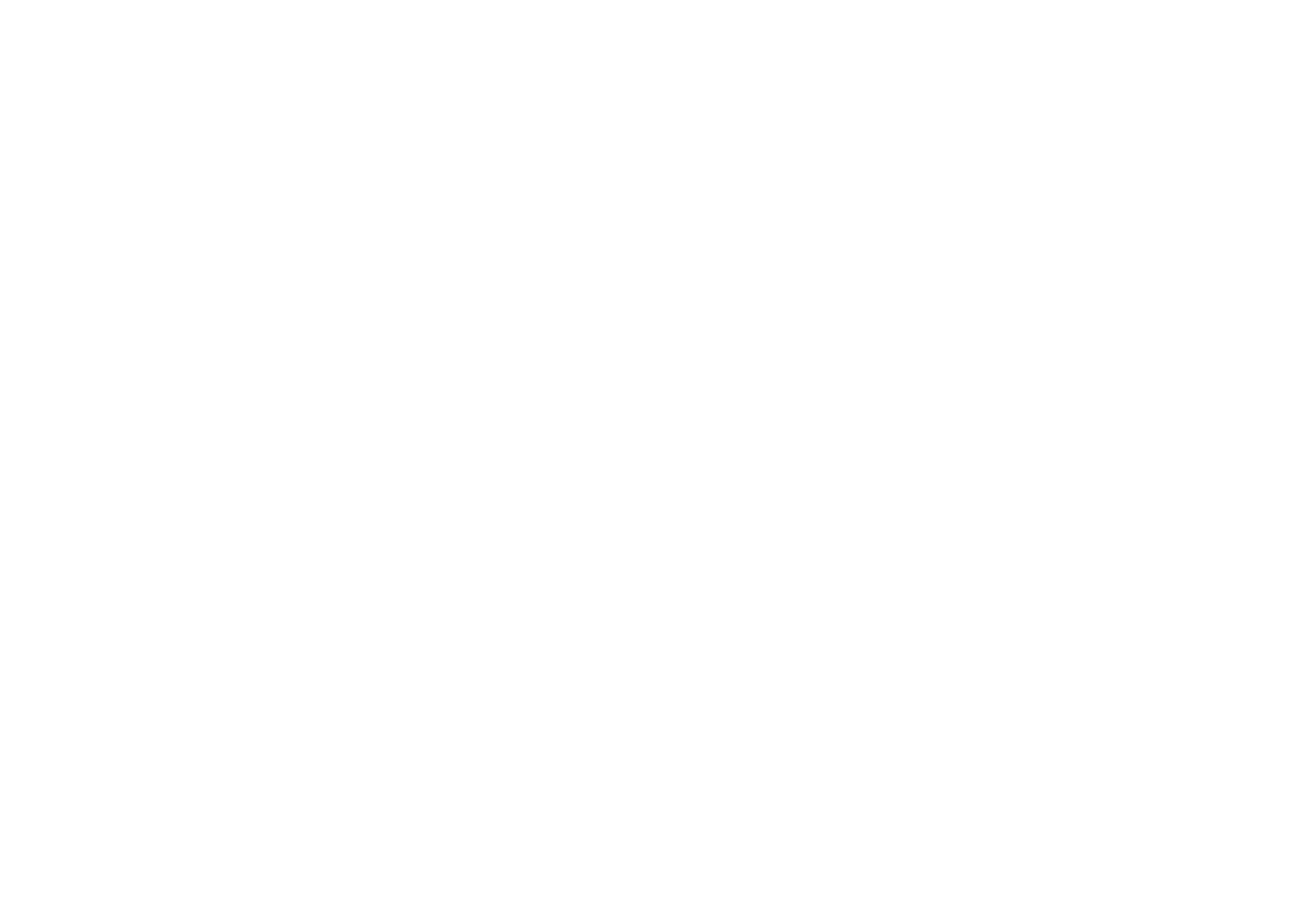

Supplement: Supplementary file 1 [file ECE3-8-9998-s001.docx]
